# Morbidity patterns among rice mill workers: A cross sectional study

**DOI:** 10.4103/0019-5278.75696

**Published:** 2010

**Authors:** Seema Prakash, Shashikala Manjunatha, C. Shashikala

**Affiliations:** Department of Community Medicine, Sri Siddhartha Medical College, Tumkur, India; 1 Department of Community Medicine, Raja Rajeshwari Medical College, Bangalore, India

**Keywords:** Cross sectional, morbidity, peak expiratory flow rate, rice mill workers

## Abstract

**Background:**

India, a land of agriculture, has formed the scaffolding for many agro-based industries. Morbidity is more common among these industrial workers; hence, this study was conducted.

**Objectives:**

To study the morbidity pattern among the rice mill workers and the relationship between duration (years) of working and their morbid status.

**Study Design:**

A cross-sectional study.

**Materials and Methods:**

A pre-structured questionnaire was used to record the necessary information such as clinical history, sociodemographic profile, findings of clinical examination and performance of peak expiratory flow rate (PEFR). The study was conducted in three rice mills in an urban area under Urban Health Training Centre (UHTC), Department of Community Medicine, SSMC, Tumkur. A total of 75 workers were recruited for the study. The study was conducted in the month of October. Statistical analysis was done using Chi-square test.

**Results:**

Among these 75 workers, 42.66% had respiratory morbidity; among them, 10.66% had PEFR less than 200L/min. 26.66% had low backache and knee joint pain and 20% had generalized / musculo skeleton pain. 6.6% suffered from allergic conjunctivitis and 4% had skin allergy.

**Conclusion:**

High Prevalence of respiratory morbidity (42.66%) and 8 (10.66%) workers with decreased PEFR were found. It also showed that there was statistically significant relationship between duration (years) of working and respiratory morbid condition. This condition can be prevented by good health education and appropriate usage of safety devices, and further studies are recommended.

## INTRODUCTION

India, being a land of agriculture, has formed scaffolding for many agro-based industries.[1] Around 58.45% of the Indian population mainly depends on agriculture for their livelihood.[2] India is the second biggest rice producing country,[1] and rice mill industry is the oldest and largest agro-based industry. Morbidity is common among the rice mill workers and their health is at risk. This made us to focus on the morbidity pattern and the working condition among the workers in an industry (rice mill).

## MATERIALS AND METHODS

The study was conducted in three rice mills in our field practicing area (Tumkur). Purposive sampling of 75 workers working of three mills was done in our study. Among them, 35 were coolies, 12 were operators, 15 were weighers, 10 were helpers and 3 were clerks. The study was conducted in the month of October 2007.

It was a cross-sectional study. A pre-structured questionnaire was used to collect the information about demographic profile, and environmental conditions of the working place, including health profile of the workers, by interview method. After detailed history taking, general physical examination was done of skin, eye, respiratory system and subjective assessment was done about musculoskeletal system. In respiratory system, chest expansion measurement and peak expiratory flow rate (PEFR) were recorded and detailed history of allergic rhinitis, cold, cough, tightness in the chest, difficulty in breathing, periodic cough and phlegm, and smoking habits was elicited.[3] Skin was examined for contact dermatitis and any allergic reactions, eyes were examined for allergic conditions, and enquiry about musculoskeletal system included low backache and joint pain.

### Analysis

Chi-square test was applied to study the relationship between duration (years) of working and their morbid conditions.

## RESULTS

The present study comprised 75 workers working in three rice mills in our field practicing area.

Table 1 shows the distribution of workers according to their age and gender. Among them, majority of the workers were in the age group of 25-35 years (43%). The mean age of the workers was 25 years and SD was 5.1.

**Table 1:** Distribution of workers according to their age and gender

Age group (years)	No (%)
15–25	20 (26.66)
25–35	32 (42.66)
35–45 and above	23 (30.66)
Total	75 (100)
Gender	
Male	60 (80)
Female	15 (20)
Total	75(100)

Males formed 80% and females formed 20%.Also, 46.66% of them were coolies and were from lower socio economic status. 16% were machine operators, 20% were weighers from lower middle class, 13.33% were helpers from lower middle class and the remaining 4% were clerks were from upper middle class.

**Figure 1:**
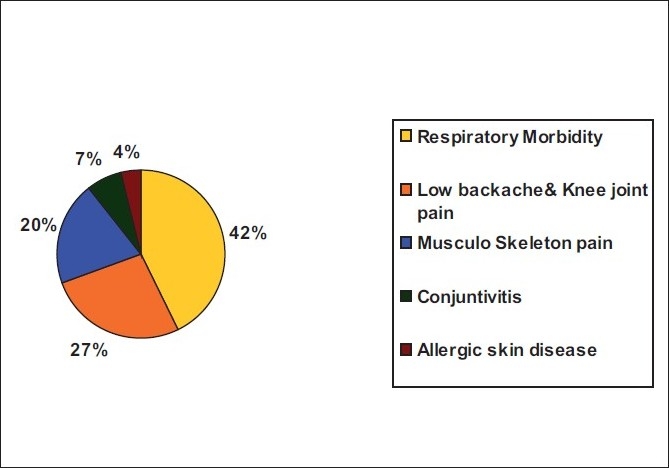
Morbidity pattern of the rice mill workers

Figure 1 shows that among the 75 workers, morbid conditions commonly affected respiratory system in 32 (42.66%), 15 (20%) had musculoskeletal pain, 12 (16%) had low backache, 8 (10.66%) had knee pain, 5 had (6.66%) conjunctivitis, followed by 3 (4%) with allergic skin diseases.

Table 2 shows PEFR among the workers. It is observed that among the 75 workers, 8 had PEFR less than 200 l/min. Among them, five were coolies and three were helpers, and these workers gave history of frequent respiratory problems like cold, cough with sputum (persistent cough with productive sputum for 2 successive years – chronic bronchitis).[3] Forty-two (53.33%) of the 75 workers had PEFR measuring between 300 and 350 l/min, followed by 13 workers having PEFR between 350 and 400 l/min and only 2 had more than 450 l/min PEFR.

Table 3 shows the relationship between the duration (years) of the work and the respiratory morbidity. Chi square (χ^2^)was applied to see the relationship between the duration of the work and respiratory morbidity and the association was found to be significant at 5% level of significance (LOS), d.f. 2, as the χ^2^ value calculated was 8.99, which was more than the χ^2^ table value of 5.99 at 5% LOS, d.f. 2.

Working place was provided with adequate lighting, ventilation and floor space area, but with inadequate toilet and eating area for the workers.

**Table 2:** Peak expiratory flow rate (PEFR) of rice mill workers

Age group (years)	No. of workers	PEFR in l/min				
		<200	250–300	300–350	350–400	>450
15–25	20	—	—	14	4	2
26–35	32	2	5	19	6	—
36–45 and above	23	6	7	7	3	—

**Table 3:** Relationship between duration of working and respiratory morbidity

Age group (years)	No. of workers	Duration of work (years)	Respiratory morbidity
15–25	20	5–7	4
26–35	32	7–9	10
36–45 and above	23	>9	18
Total	75	—	32

## DISCUSSION

The study revealed that prevalence of respiratory morbidity [32 (42.66%)] was significantly higher among the workers and the prevalence of chronic bronchitis could be attributed to occupational exposure to dust and smoking habits among the rice mill workers and also the duration (years) of working. Other important morbidities included low backache and knee joint pain, which was found in around 20 (26.66%) workers, followed by musculoskeletal pain in 15 (20%), conjunctivitis and allergic skin diseases.

These findings are in agreement with those of the study by Meo *et al.,*[4] in which a significant reduction of forced expiratory volume in 1 second (FEV_1_), forced vital capacity (FVC), peak expiratory flow (PEF) and maximal voluntary ventilation (MVV) was found among the workers who worked more than 5-8 years, and similarly, the study of Musa *et al.,*[5] which showed significant decrease in FEV_1_ and FVC among the workers who worked for more than 11 years.

Ye *et al.*[6] showed that rice granary workers had lower mean FEV/FVC values both pre and post shift, which showed that there was an association between chronic grain-dust exposure and chronic airway obstruction.

Bhat *et al.’*s[7] study showed that there was comparative decrease in PEFR/min within 1 year after the workers joined the job. Pradhan *et al.,*[8] in their study, found that 59% suffered from knee joint pain and 61.5% suffered from low back and knee joint pain. Karim *et al.*[3] studied the prevalence of allergy and conjunctivitis, which revealed allergic symptoms in 17% and conjunctivitis in 19%.

## CONCLUSIONS

The important morbidities detected among the rice mill workers were chronic bronchitis, allergic bronchitis, low backache and knee joint pain. Even with adequate ventilation and lighting at the working place, the respiratory morbidity was quite significant among these workers, which might be attributed to occupational exposure to dust among the workers. These findings are in agreement with those of other studies conducted on these workers. Based on the findings, we recommend the following.

- Further studies with large sample size.
- The use of protective devices such as respiratory masks and goggles by these workers should be promoted in order to reduce the occupational exposure to dust.
- Occupational health services should be provided to these workers, which include pre-placement examination, routine health check-ups, health education and rationalization of the work methods so as to improve the health and safety of the workers.
